# Lengths of Orthologous Prokaryotic Proteins Are Affected by Evolutionary Factors

**DOI:** 10.1155/2015/786861

**Published:** 2015-05-31

**Authors:** Tatiana Tatarinova, Bilal Salih, Jennifer Dien Bard, Irit Cohen, Alexander Bolshoy

**Affiliations:** ^1^Children's Hospital Los Angeles, Keck School of Medicine, University of Southern California, Los Angeles, CA 90027, USA; ^2^Department of Evolutionary and Environmental Biology and Institute of Evolution, University of Haifa, 3498838 Haifa, Israel; ^3^Department of Computer Science, University of Haifa, 3498838 Haifa, Israel; ^4^The Tauber Bioinformatics Research Center, University of Haifa, 3498838 Haifa, Israel

## Abstract

Proteins of the same functional family (for example, kinases) may have significantly different lengths. It is an open question whether such variation in length is random or it appears as a response to some unknown evolutionary driving factors. The main purpose of this paper is to demonstrate existence of factors affecting prokaryotic gene lengths. We believe that the ranking of genomes according to lengths of their genes, followed by the calculation of coefficients of association between genome rank and genome property, is a reasonable approach in revealing such evolutionary driving factors. As we demonstrated earlier, our chosen approach, Bubble-sort, combines stability, accuracy, and computational efficiency as compared to other ranking methods. Application of Bubble Sort to the set of 1390 prokaryotic genomes confirmed that genes of Archaeal species are generally shorter than Bacterial ones. We observed that gene lengths are affected by various factors: within each domain, different phyla have preferences for short or long genes; thermophiles tend to have shorter genes than the soil-dwellers; halophiles tend to have longer genes. We also found that species with overrepresentation of cytosines and guanines in the third position of the codon (GC_3_ content) tend to have longer genes than species with low GC_3_ content.

## 1. Introduction 

To better understand the interaction between the environment and bacteria, whether in a human host or any other ecosystem, one must know the laws governing prokaryotic evolution and adaptation to environment. For example, it is essential to study how a change in pH or external temperature affects a bacterial genome and especially its coding sequences. Unfortunately, the laws of prokaryotic coding sequence evolution remain unclear. Orthologous proteins may drastically differ in both codon usage and length across species. When a gene length changes, a protein may acquire a new function or lose an existing one, hence, changing the entire ecosystem. Many studies have analyzed the relationship between codon usage and the environment [1–3], but a few efforts were made to predict the effect of a changing environment on gene length. The main results were related to comparative analysis between protein lengths in eukaryotes and prokaryotes. Detailed comparison of protein length distributions in eukaryotes and prokaryotes can be found in [4, 5]. Wang et al. [6] proposed that “molecular crowding” effect and evolution of linker sequences can explain differences between length of orthologous sequences in super-kingdoms. Our study is focused on protein lengths in prokaryotes, exclusively.

How does gene length change occur in prokaryotes? The main driving force in shaping gene length is a point mutation [7]. Point mutations may cause a stop codon shift, when the existing stop codon is destroyed and gene length is increased, a start codon drift, or appearance of a premature stop codon. To understand trends of fixation of mutations changing protein lengths we performed a comparative study of lengths of paralogs. We explore the use of seriation of genomes based on paralogs' lengths.

In recent papers [8, 9], we formulated the genome ranking problem, listed several approaches to solve it, described a novel method for genome ranking according to gene lengths, and demonstrated preliminary results from the ranking of prokaryotic genomes. These results indicated that hyperthermophilic species have shorter genes than mesophilic organisms. We hypothesize that gene lengths are not randomly distributed; instead they are affected by a number of environmental, genomic and taxonomic factors. In this paper we present a framework for analysis of gene lengths and evaluate effects of environmental factors.

In order to analyze evolutionary pressures acting on genes it is necessary to group them into well-defined functional categories. There are several existing approaches. First of all, there is the most popular database of Clusters of Orthologous Groups (COG) of proteins, which is a comprehensive collection of prokaryotic gene families. This database was created to classify the complete complement of proteins encoded by complete genomes based on evolutionary development. The data in COGs are updated continuously following the sequencing of new prokaryotic genomic sequences. As described by Tatusov et al. [10], the COGs database is a growing and useful resource to identify genes and groups of orthologs in different species that are related by evolution. Sixteen years ago, the database was started with only seven Bacterial genomes; in 2010 the database consisted of proteins from 52 Archaeal and 601 Bacterial genomes (a total of 653 complete genomes) that were assigned to 5,663 COGs; currently it contains approximately 2 K genomes.

The COG database is not the only possible data compilation to classify prokaryotic proteins. Since its publication over a decade ago, additional classifications have appeared. In 2007,* Archaea* were grouped into the acCOG database [11]. Another alternative, the eggNOG database [12, 13], grouped gene families at the universal level, covering all three domains of life.

Recently, Bolshoy et al. introduced a “gene-length based” model [14, 15], representing genomes as vectors of genes. The set of genomes is represented as a matrix, in which each row stands for a genome and each column stands for a gene family. Therefore, each element of this matrix stands for the length of a member of a gene family *i* in a genome *j*. In our study, the objects are annotated prokaryotic genomes; the descriptors are the lengths of the genome proteins indexed according to the COG database.

A ranking is a relationship between a set of objects such that, for any two objects, the first is either ranked “higher than,” “lower than,” or “equal to” the second. Gene ranking is a useful approach to answer biological questions, however it is sometimes difficult to implement. Here we bring examples of usage this measure in biologic sciences. A prioritization or ranking is used in bioinformatics to aid in the discovery of disease-related genes. Computational methods are employed for ranking the genes according to their likelihood of being associated with the disease. A variety of methods have been conceived by the researchers for the prioritization of the disease candidate genes. A review of various aspects of computational disease gene prioritization and related problems is presented in Gill et al. [16].

In our case, the goal is to order the genomes that are represented as rows of a gene length matrix. There are different possible approaches to define the optimal rank of rows in the matrix. We have previously determined [9] that Bubble Sort method (B-Sort, see Section 5) is more accurate than Average Sort and Simple Additive Ranking and it is as accurate and significantly faster than the Simulated Annealing Procedure.

The complexity of the ranking problem using matrices with missing values was discussed in detail [17]. The same ranking problem appears in several areas of operations research, such as in the context of group decision making [18] and country-credit risk rating [19]. Missing data as well as variable relative importance of different gene families make the problem increasingly complex. To the best of our knowledge, genome ranking problem has been addressed for the first time in [8, 9].

Establishing ordered lists of genomes using lengths of coding sequences of orthologous genes, we aim to find an association between a genome rank and a genome property of interest, such as its role in virulence and adaptation. There are many different types of such properties: a prokaryote can be either Archaea or Bacteria; an organism may be hyperthermophile, thermophile, psychrophile, or mesophile; a genome has a certain GC-content, and so on. In summary, the goal is to find out whether gene lengths of a genome are associated with various genome properties and to measure the magnitude of this association. These findings will allow us to determine important factors such as virulence, biofilm formation, and antimicrobial resistance that may be associated with the pathogenesis of a specific species and the ability to cause serious infections in patients.

## 2. Results 

We used a dataset of 1390 genomes (the “big” dataset) and a randomly selected subset of 100 genomes (the “small” dataset). For each dataset we used complete and filtered versions. The filtering procedure (see Section 5) removes those COGs that are present in only a small number of genomes and are likely to skew the ordering results. We set the frequency threshold to be 35%, meaning that the filtering procedure removes COGs present in less than 35% of analyzed genomes. After filtering we obtained the filtered dataset containing 1474 COGs.

**Figure 1:**
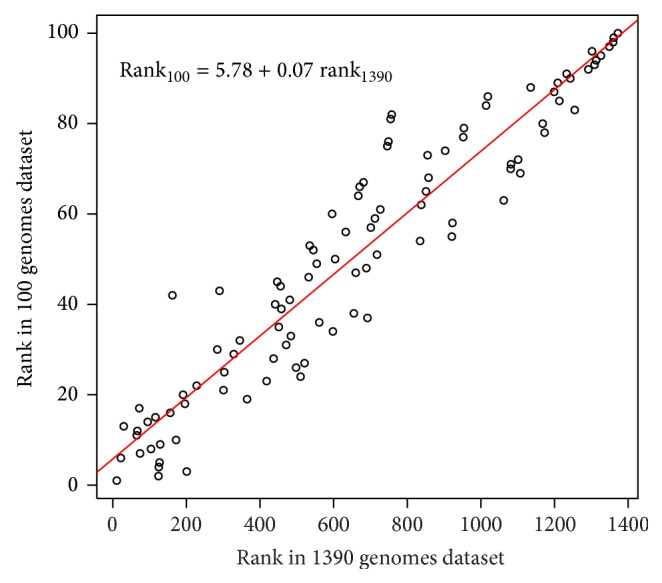
Consistency of Bubble Sort ranks in 1390 and 100 genomes datasets. Pearson's correlation coefficient between two ranks is 0.95; Kendall tau correlation coefficient is 0.82.

We assessed the consistency of ranks of genomes of the small dataset in two orderings: of the entire collection of 1390 genomes and of the subset of 100 genomes (Figure 1). We determined corresponding ranks of 100 genomes in the B-sorted dataset of 1390 genomes and discovered that the two orderings of 100 genomes were highly consistent (with correlation coefficient of 0.95). This confirms that the ranking procedure is stable. However, random selection of a small subset may cause wrong ranks of a few isolated genomes. Indeed, there are some genomes that show differences in 100 and 1390 genomes rank, for example, bacteria* Sodalis glossinidius*, which is ranked 42 in 100 genomes and 162 in 1390 genomes dataset. Therefore, the ranks' consistency found for the huge majority of ranks is an additional support to the chosen method of ranking.

**Figure 2:**
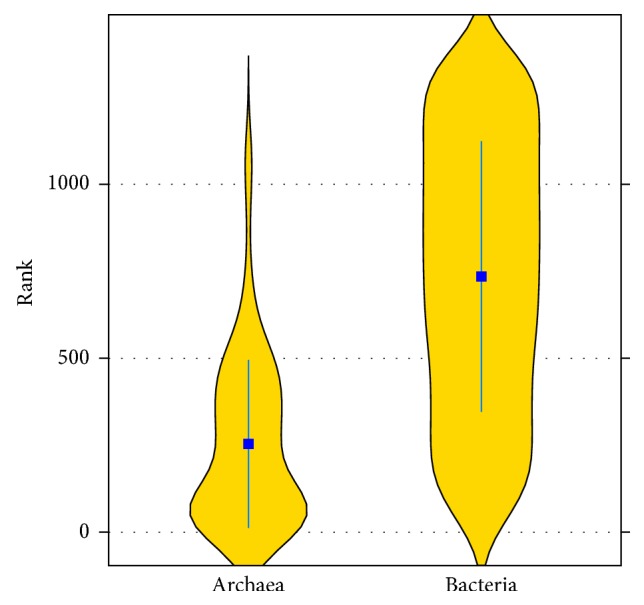
Violin plots of Bubble sort ranks of Archaea and Bacteria. Average rank of 1276* Bacterial* genomes is 735 and average rank of 114* Archaeal* genomes is 254.

Let us start with an overview of the orderings; let us compare ranks of Bacteria and Archaea. (Larger value of a genome rank means longer genes in this genome.) Bacterial genomes have a broader distribution of ranks than Archaeal genomes (Figure 2). Overall, Bacterial ranks are larger than Archaeal ranks in the 1390 genome, as well as in 100 genome datasets. This observation can be illustrated using the violin plot of ranks' distributions, as shown in Figure 2. Average rank of 1276* Bacterial* genomes was 735 and average rank of 114 Archaeal genomes was 254. This visual observation is also supported by a simple statistical procedure. Using the Wilcoxon rank test and *α* = 0.01, we calculated the test statistic *T*_*a*_, equal to the sum of the ranks for the ordered data that belong to Archaea. *T*_*a*_ was 28,913. For large samples *T*_*a*_ is approximately normal with expected value and standard deviation calculated as

(1) $$\begin{array}{c} E\left(T_{a}\right)=\frac{n_{a}\left(n_{b}+n_{a}+1\right)}{2}=79,287,\\ \sigma \left(T_{a}\right)=\sqrt{\frac{n_{a}n_{b}\left(n_{a}+n_{b}+1\right)}{12}}=4106.3. \end{array}$$

Therefore,

(2) $$\begin{array}{c} Z=\frac{T_{a}-E\left(T_{a}\right)}{\sigma \left(T_{a}\right)}=-12.27,\\ P\left(Z<-12.27\right)\approx 10^{-34}<0.01. \end{array}$$

Hence, we conclude that* Bacterial* genomes rank significantly higher than* Archaeal* genomes. Tables 1 and 2 show the summary statistics for the ordering of Archaeal and Bacterial genomes. These tables show mean, median, range, and standard deviation of Archaeal and Bacterial ranks of 1390 genomes stratified by phylum. In the Bacterial domain, Firmicutes and Thermotogae have shorter genes and Actinobacteria have longer ones. In the Archaeal domain, Euryarchaeota have longer genes than Crenarchaeota. These results are consistent with our earlier findings from analysis of 100 prokaryotic genomes [8].

Next, we considered the nucleotide composition of coding regions. In prokaryotes, the nucleotide composition of coding regions varies significantly between species. GC_3_ (frequency of cytosine and guanine in the third position of the codon) is one of the variable features. Across the Bacterial domain, GC_3_ ranges from 10% to 90% [20]. Tatarinova et al. previously demonstrated [21, 22] that, within one eukaryotic species, GC_3_ content can be used to distinguish two classes (housekeeping and stress-specific) genes. Currently, we sought to evaluate mutation pressure acting on the entire prokaryotic genome by examining how the average GC_3_ content, calculated across all genes in a genome, is related to the position of the genome in a global ordering. We calculated the GC_3_ content of coding regions across all analyzed genomes and discovered that the genome rank and cytosine/guanine content of the third codon position of genes are positively correlated (Spearman rank correlation coefficient *ρ*(GC_3_, rank) = 0.62 for* Bacteria* and *ρ*(GC_3_, rank) = 0.59 for* Archaea*). For example, the average GC_3_ content in Actinobacteria (0.70) is twice the amount seen in Firmicutes  (0.35).

## 3. Discussion 

The ability of some species to grow at high temperatures has been a long-term fascination of microbiologists. Proteins of hyperthermophilic species are more resilient to heat and are shorter than proteins of mesophilic species. Understanding this effect is important for biotechnology [23].

Up to now, less than a dozen studies were devoted to protein length distribution. Among those, there were only four relevant publications: [4, 5, 8, 24]. In 2000, using an early version of the COG database, Zhang compared 22 species in three domains of life [4] and found that the average gene length is smallest for* Archaea* and greatest for eukaryotes. Similarly, Skovgaard et al. [24] analysed 34 prokaryotic genomes and discovered that, for the vast majority of functional families, Bacterial proteins were longer than* Archaeal* ones. In 2005, Brocchieri and Karlin [5] confirmed these findings using a larger collection of genomes (16* Archaeal* and 67* Bacterial* species). They found that bacteria were enriched in functional families with longer genes. In addition, they described a negative correlation between protein length and optimal growth temperature of* Archaea* and* Bacteri*a. By grouping proteins into broad functional classes (information storage and processes; cellular processes; metabolism; poorly characterized; not characterized) and comparing their median lengths, Brocchieri and Karlin concluded that “information storage and processes” proteins are shorter than “cellular processes” and “metabolism” proteins. They also found that Archaea have more of the shorter and poorly characterized proteins.

The above mentioned studies, performed on relatively small sets of genomes, share the same deficiency of using average (mean or median) lengths of genes in a genome to reach their conclusions. As we illustrated in [8, 9] this approach can substantially distort results. In [8, 9] we proposed a systematic framework to analyse the relationship of prokaryotic gene lengths and environmental conditions that is not based on analysis of average lengths of proteins. This framework, further investigated in the current paper, allows more flexibility and produces more meaningful results than the previous approaches.

Hyperthermophilic species of Archaea and Bacteria, living in extreme environments (such as volcanic hot springs) occupy the top portions of the ranking lists of the small and big datasets. At a first glance it appears that we could hypothesize that extremophiles have shorter genes than species living under normal conditions. However, the situation appears to be more complex. For illustration we consider Sulfolobales, Thermoproteales, and Halobacteriales. Sulfolobales grow in volcanic hot springs at pH 2-3 and a temperature of 75–80 degrees Celsius. In the ordered list of 1390 genomes, Sulfolobales occupy positions from 12 to 94, which means that as a rule Sulfolobales have very short genes.* Thermoproteales* (extremely thermoacidophilic anaerobic Archaea isolated from Icelandic solfataras) also have very short genes, their genomes are found in positions from 7 to 77 but also in positions from 412 to 460 in the ordered list, which are positions of genomes with moderately short genes. Halobacteriales (found in water saturated or nearly saturated with salt) are placed in positions from 541 to 1263 which are not considered genomes with short genes. From these observations follows that stress of living in* an arbitrary* extreme environment is not the factor, while, probably, hyperthermophilicity and halophilicity are the factors affecting orderings in opposite directions.

We also showed that, as a group, Bacterial genomes are ranked significantly higher than the Archaeal ones according to the length of their genes (Figure 2). This observation may be explained by the fact that the vast majority of completely sequenced Archaeal genomes are hyperthermophiles, which tend to have shorter genes as compared to psychrophiles and mesophiles. Our previous speculations obtained on relatively small datasets [8] and our current results on 1390 genome dataset are consistent with the hypothesis that high temperature environment is a factor causing reduction of gene length. In the 100-genome dataset hyperthermophiles occupy positions in the top portion of the list: top 20 in the 100 genomes list. They are also ranked in the top of the 1390 genome dataset.

We also observed that 34% of the shortest (first 100 positions in the ordered list) of 1390 genomes are occupied by hyperthermophilic species, while none are found in the longest (last 100 in the ordered list). Furthermore, 90% of thermophiles are placed in the top third of the list. Moderately thermophilic species are not restricted to the top positions. For example,* Thermobifida fusca* (a moderately thermophilic soil bacterium growing at 55°C and a major degrader of plant cell walls in heated organic materials such as compost heaps, rotting hay, manure piles or mushroom growth medium) occupies position 1260 in the ordered list.* Anaerolinea thermophila*, with similar growth temperature, has a close position of 1173.

There are several remarkable features that appeared as a result of the 1390 genome ordering. Campylobacterales (belonging to the phylum Proteobacteria) have an average position of 203, with the smallest position of 10 (*Helicobacter bizzozeronii ciii-1*) and the largest position of 392 (*Helicobacter hepaticus atcc 51449*). Most species in this family are human and animal pathogens. Namely,* Campylobacter jejuni* is a microaerophilic bacterium frequently associated with gastroenteritis in humans. Complications such as meningitis [25], septicemia [26], and Guillain-Barré syndrome have also been reported [27]. In addition,* Helicobacter bizzozeronii* (position 10) has been implicated in gastric infections, similar to* Helicobacter pylori*, referred to as* non*-*Helicobacter pylori Helicobacter* (NHPH) infections in humans [28]. It appears that all known Campylobacterales have short genomes. It is tempting to speculate that there are evolutionary pressures to keep genes in short pathogenic genomes as short as possible.

However, not all pathogens have short genes. Not even all pathogens with short genomes have short genes. Common obligate intracellular prokaryotic pathogens from the phylum of Chlamydiae are very small (measuring 0.3–0.6 *μ*m in diameter) and grow by infecting eukaryotic host cells. This phylum is comprised of several major intracellular pathogens of humans and animals, causing a variety of diseases. These bacteria can cause keratoconjunctivitis, pneumonitis, and sexually transmitted infections. In spite of its small physical dimensions, Chlamydiae have exceptionally long genes: the ranks of 21 members of this class are located from positions 835 to 1127 in the ranking list. We speculate that there are certain evolutionary factors (yet to be discovered) that keep Chlamydiae genes so long.

As we see, Campylobacterales (of the phylum Proteobacteria), have short genes. At the opposite end of the length spectrum we find the phylum Actinobacteria, tending to have longer genes. Only 8 out of 137 species of Actinobacteria have positions below 1000 in the ordered set. One of the species, a pathogenic bacterium* Renibacterium salmoninarum* [29], was placed among species with characteristically short genes in the position 343. The genome of* R. salmoninarum* has extended regions of synteny to the* Arthrobacter* sp. strain FB24 and* Arthrobacter aurescens* TC1 genomes, but it is approximately 1.9 Mb smaller than two sequenced* Arthrobacter* genomes and has a lower GC content [29]. In the Bubble Sort list,* Arthrobacters* occupy positions 1230, 1301, 1342, 1343, and 1354. Our results show that significant genome reduction, which has occurred since divergence from the last common ancestor, affected not only gene content but also lengths of remaining genes. It is possible that factors affecting gene lengths of Actinobacteria are different from the factors acting on Chlamydiae, while resulting in keeping proteins longer in both cases.

Relationships between gene length and codon bias have been previously studied by [30–33]. Oliver and Marín [30] and Xia et al. [32] observed a positive correlation between length and GC composition of coding sequences in prokaryotes, attributing the effect to reduced frequency of stop codons in GC-rich species. Later Xia et al. [33] mentioned that the correlation is weak for a number of species, with 4 species showing a negative correlation. Thus Xia et al. formulated a more general hypothesis incorporating selection against cytosine (C) usage. In [33] they described two additional factors giving rise to this selection: transcription efficiency and “insurance” against cytosine deamination.

Third positions in codon are largely degenerate; 70% of changes at third codon positions are synonymous [34]. Therefore, it makes sense to analyze adaptation effects using GC composition in the third position of the codon, GC_3_. We showed that adaptation to higher temperatures affects the genome in two ways: first, GC_3_ content of genes tends to increase with growth temperature [35]; at the same time, hyperthermophilic species tend to have shorter genes as it can be seen from the ranks of these species both in the 100-genome dataset and in the larger dataset. Several factors may compete for placement of the Bacterial species in the ordering rank. Adaptation to high temperatures and pathogenicity may tend to place an organism into lower ranks. High GC_3_ composition and adaptation to high salinity environments places an organism into higher ranks. However, future research is needed to determine important factors, both environmental and genomic, that may affect the rank of the genome. This information will allow us to further understand and possibly predict the invasive or virulent nature of a particular species compared to a nonpathogenic organism that is part of the normal commensal flora of an individual. Further exploration of these factors may also answer questions on the emerging mechanisms of resistance that may be associated with specific organisms and on prediction of resistance using novel methods other than conventional susceptibility tests.

We will continue updating our collection of prokaryotic genome orderings. When a new genome is sequenced, it is not necessary to repeat the entire ranking procedure from an unordered dataset. In order to incorporate a newly sequenced genome in our analysis, it is necessary to (1) predict genes and (2) assign COG categories. Then the new, completely annotated, genome can be added to the presorted data matrix, using average gene length as a rough indicator of the new genome position. Then the ranking procedure should be applied to the updated matrix. Since all but one of the genomes is already in the correct place, the ranking procedure will have to make only a small number of steps to determine the rank of a new genome.

## 4. Conclusions

We applied Bubble Sort to the set of 1390 prokaryotic genomes and revealed several interesting trends. We demonstrated that hyperthermophiles may be always characterized as having short proteins. Also, the resulting ordering showed that Archaea have shorter genes than Bacteria, and we speculate that this can be attributed to the prevalence of hyperthermophiles among the sequenced Archaea. Within each domain, different phyla have preferences for short or long genes. Another interesting observation is the significant correlation between gene length and GC composition of coding regions. Therefore, we suggest that gene lengths are not randomly distributed across species but are shaped by environmental and genomic factors.

The genome ranking procedure is stable. Inclusion of additional genomes does not distort the relative ranking of genomes. The correlation coefficient between the ranks of the 100 genomes in the 100-genome dataset and in the larger (1390) dataset is 0.95. Hyperthermophilic species are ranked on top in both 100 and 1390-genome lists; soil dwelling species are consistently at the bottom of the list.

Our results show that environmental factors constitute a strong force that groups evolutionary distant species together in protein-lengths' ranking. On the other hand, evolutionary history and phylogenetic closeness group certain organisms together as well. Relative influence of these factors varies between organisms. For example, we demonstrated that hyperthermophilic species have shorter genes than mesophilic organisms, which implies that environmental factors may affect gene length. However, not every environmental stress has the gene shortening effect. For example, high salinity represents an extreme environment that relatively few organisms have been able to adapt to and occupy. Halophiles are a type of extremophile organisms that live in high salt concentrations. Seemingly, high salinity opposite to high temperature does not cause protein-length decrease; the extreme halophiles (or halobacteria), tend to have pretty long genes.

## 5. Materials and Methods

All four ranking algorithms discussed in this paper were applied to input matrices based on the database of Clusters of Orthologous Groups of proteins (COG) [10, 36–38]. As of October 2012, there were 5664 COGs, 1276 Bacterial and 114 Archaeal genomes sequences in the NCBI database. The sequences were processed according to the procedures described below.

### 5.1. COGs Database

Information about every completely sequenced and annotated prokaryotic genome is stored as tables of protein features, called PTT files, prepared by the National Center for Biotechnology Information (NCBI). The complete collection of current PTT files can be found at ftp://ftp.ncbi.nih.gov/genomes/.

From every prokaryotic NCBI PTT file, we extracted information about each gene length, COG and added the genome index (tax id). We created a combined gene-length matrix, where rows correspond to genomes, identified by taxonomy id, and columns correspond to COGs. Each element (*i*, *j*) of this matrix is a length of gene belonging to COG *i* in genome *j*. All currently available genomes were described in these two files. To check the ranking methods described below we used small subsets (100 genomes) of this dataset.

### 5.2. Preprocessing Procedures

To get an input file for further ranking the following preprocessing procedures developed by Bolshoy et al. [9, 15, 39] were applied.

1. *Selection of Genome Subsets*. A subset may be defined applying different criteria: it may be either a representative sample, a taxaspecific subset, or randomly chosen genomes.
2. *Application of a Filtering Parameter (An Entry Threshold) on a Selected Subset*. Only COGs containing more than a threshold number of genomes are considered for further processing. For example, if the filtering value is equal to 20% and an amount of genomes in a subset is equal to 500, then only COGs containing at least 100 genomes are considered (passed the entry threshold).
3. *Sampling*. If there are multiple instances of a COG related to the same genome, a median length value for all paralogs from the same genome and from the same COG is used for further processing.

### 5.3. Sets of Genomes

As of May 2012, there were approximately 1500 NC-numbers, corresponding to 1390 annotated prokaryotic genomes at NCBI. Multiple NC numbers occur for prokaryotes with more than one chromosome, such as* Burkholderia cepacia* (Tax id 269483). This large set was used for the final Bubble sort analysis. In that set, 114 genomes are Archaeal and 1276 are Bacterial. To compare performance of the methods, we used a small subset of this dataset, same as we used previously [8]. Then, we had randomly selected 100 prokaryotic genomes out of a possible 1390, contained at the NCBI COG database. This small set contains 9 Archaeal and 91 Bacterial genomes. The list of selected genomes is shown in Table 3. After the selection of genomes, we discarded those COGs that were present in less than 35% of those selected genomes. Upon filtering, our input contained 1455 COGs. Note, that the input file is a sparse matrix.

### 5.4. Bubble Sort Ranking (B-Sort)

As a LOPI strategy [40] we apply here the regular “bubble sort” procedure [41] interchanging the rows of a given matrix. (In a simulation study on graphs [42], the LOPI strategies found a global maximum of the goal function defined on edges in the majority of the cases.) The criterion by which the procedure decides whether rows would be interchanged is as follows. Comparing two genomes we take into account only those COGs that both genomes have members in them. Comparing pairs of lengths of genes from relevant COGs we count which genome in a pair has longer genes more frequently. In other words, if a genome associated with a row *i* has longer genes than has a genome associated with a row *i* + 1, then these rows would be interchanged. We note that due to application to a sparse matrix this procedure would not necessarily lead to the optimal ordering.

### 5.5. Solving of the Optimization Problem

The three methods above are pretty intuitive. They do not have a goal to find an optimal ranking but the results have a good chance to be close to the optimal ranking. In our review [8] we described several procedures to find a nearly optimal ranking using approach from the field of combinatorial optimization. Maximization of an average Kendall tau rank correlation coefficient is one of them. As we presented it, the goal is to assign each genome *i* to a scale *x* such that *x*_*i*_ most accurately recovers the across-genome gene lengths. “Most accurately” here means achieving the maximum of the function *x* ^*τ*^:

(3) $$\begin{array}{c} x^{\tau }=\underset{x}{\max}\left[\overset{K}{\underset{k=1}{\sum }}\overset{N-1}{\underset{i=1}{\sum }}\overset{N}{\underset{j=i+1}{\sum }}C_{ij}\left(\vec{x},\vec{r^{k}}\right)\right], \end{array}$$

where given a rating vector $\vec{x}$ and an “individual” vector $\vec{r^{k}}$ of the gene lengths of COG *k*, $C_{ij}(\vec{x},\vec{r^{k}})$ is equal to 1, if (*r*_*x*_*i*__^*k*^ < *r*_*x*_*j*__^*k*^), equal to 1/2, if (*r*_*x*_*i*__^*k*^ = *r*_*x*_*j*__^*k*^), and 0-otherwise.

### 5.6. Kemeny-Optimal Ranking

Kemeny-Optimal Ranking is an* optimal rank aggregation* approach. In [43, 44] the authors proposed a precise criterion for determining the “best” aggregate ranking. Given *n* objects and *k* permutations of the objects, {*π*_1_, *π*_2_,…, *π*_*k*_}, a* Kemeny optimal* ranking of the objects is the ranking *π* that minimizes a “sum of distances” $P=\sum_{i=1}^{k}d(\vec{x},\vec{r^{k}})$, where $d(\vec{x},\vec{r^{k}})$, denotes a distance between a rating vector $\vec{x}$ and an “individual” vector $\vec{r^{k}}$ based on* Kendall's* **τ** *rank-correlation*. From the properties of Kendall's **τ** rank-correlation it follows that a Kemeny optimal ranking minimizes the number of pairwise* disagreements* with the given *k* rankings *x* ^*τ*^ and maximizes sortedness.

It is known that finding a Kemeny optimal ranking is NP-hard [45] and remains NP-hard even when there are only four input lists to aggregate [46]. This motivates the problem of finding a ranking that* approximately* minimizes the number of disagreements with the given input rankings.

## Figures and Tables

**Table 1:** B-sort results (one run) for 1390 genomes, archaea.

Phylum	Average rank	StDev	Median rank	Rank range	Number of genomes
Crenarchaeota	189	179	77	7–492	35
Euryarchaeota	312	297	233	5–1263	74
Korarchaeota	169	NA	169	169–169	1
Nanoarchaeota	5	NA	5	5–5	1
Thaumarchaeota	347	239	347	178–516	2
Unclassified archaea	771	NA	771	771–771	1

**Table 2:** B-sort results for 1390 genomes, bacteria.

Phylum	Average rank	STD	Median rank	Rank range	Number of genomes
Actinobacteria	1223	166	1260	343–1390	137
Aquificae	182	79	168	82–306	8
Bacteroidetes/Chlorobi	992	188	1071	502–1359	71
Candidatus Cloacamonas	1054	NA	1054	1054–1054	1
Chlamydiae/Verrucomicrobia	1076	81	1079	835–1223	25
Chloroflexi	774	520	1109	70–1274	15
Chrysiogenetes	545	NA	545	545–545	1
Cyanobacteria	938	209	975	619–1276	40
Deferribacteres	205	NA	205	205–205	1
Deinococcus-Thermus	607	282	566	263–1126	12
Dictyoglomi	207	49	207	172–242	2
Elusimicrobia	412	143	412	311–513	2
Fibrobacteres/Acidobacteria	1171	172	1240	839–1293	6
Firmicutes	307	188	286	21–1387	271
Fusobacteria	462	100	461	361–564	4
Gemmatimonadetes	1214	NA	1214	1214–1214	1
Nitrospirae	563	418	563	267–858	2
Planctomycetes	1364	29	1368	1319–1389	5
Proteobacteria	759	325	775	1–1379	588
Spirochaetes	1050	155	1066	700–1317	31
Synergistetes	466	40	466	438–494	2
Tenericutes	657	223	631	92–1092	36
Thermobaculum	1049	NA	1049	1049–1049	1
Thermodesulfobacteria	458	32	458	435–480	2
Thermotogae	253	165	203	45–566	12

**Table 3:** List of Archaeal (A) and Bacterial (B) genomes in the Bubble Sort ordering rank, 100 genomes dataset. Hyperthermophiles, Streptococci, and Enterococci are marked in the Note column.

Rank	Domain	Note	Organism
1	A	Hyperthermophile	*Archaeoglobus fulgidus dsm 4304 *
2	A	Hyperthermophile	*Thermoplasma volcanium gss1 *
3	B	Hyperthermophile	*Thermotoga sp. rq2 *
4	A	Hyperthermophile	*Thermoplasma acidophilum dsm 1728 *
5	B	Hyperthermophile	*Thermotoga neapolitana dsm 4359 *
6	A	Hyperthermophile	*Thermococcus onnurineus na1 *
7	B		*Campylobacter concisus 13826 *
8	B		*Campylobacter curvus 525.92 *
9	B	Hyperthermophile	*Aquifex aeolicus vf5 *
10	B	Hyperthermophile	*Dictyoglomus thermophilum h-6-12 *
11	B		*Bacillus cereus atcc 14579 *
12	B		*Bacillus cytotoxicus nvh 391-98 *
13	B		*Melissococcus plutonius atcc 35311 *
14	A	Hyperthermophile	*Thermococcus sibiricus mm 739 *
15	B		*Listeria monocytogenes clip81459 *
16	B		*Bacillus amyloliquefaciens dsm 7 *
17	B		*Rickettsia canadensis str. Mckiel *
18	A	Hyperthermophile	*Pyrococcus abyssi ge5 *
19	B		*Helicobacter felis atcc 49179 *
20	A	Hyperthermophile	*Pyrococcus horikoshii ot3 *
21	B	Streptococcus	*Streptococcus pneumoniae p1031 *
22	B	Streptococcus	*Streptococcus agalactiae a909 *
23	B		*Caldicellulosiruptor bescii dsm 6725 *
24	B		*Mycoplasma fermentans m64 *
25	B	Streptococcus	*Streptococcus agalactiae 2603v/r *
26	A		*Methanosalsum zhilinae dsm 4017 *
27	B		*Francisella sp. tx077308 *
28	B	Streptococcus	*Streptococcus equi subsp. zooepidemicus *
29	B		*Bacillus pumilus safr-032 *
30	B		*Pediococcus pentosaceus atcc 25745 *
31	B		*Geobacter lovleyi sz *
32	B	Enterococcus	*Enterococcus faecalis v583 *
33	B		*Natranaerobius thermophilus jw/nm-wn-lf *
34	B		*Mycoplasma pulmonis uab ctip *
35	B		*Brevibacillus brevis nbrc 100599 *
36	B		*Mycoplasma genitalium g37 *
37	B		*Mycoplasma leachii pg50 *
38	B		*Ureaplasma parvum serovar 3 *
39	B		*Bacillus thuringiensis str. al hakam *
40	B		*Neisseria meningitidis 053442 *
41	B		*Legionella pneumophila str. paris *
42	B		*Sodalis glossinidius str. “morsitans” *
43	B		*Candidatus riesia pediculicola usda *
44	B		*Lactobacillus gasseri atcc 33323 *
45	B		*Coxiella burnetii rsa 331 *
46	B		*Laribacter hongkongensis hlhk9 *
47	B		*Ruminococcus albus 7 *
48	B		*Mycoplasma pneumoniae m129 *
49	A		*Halalkalicoccus jeotgali b3 *
50	B		*Geobacter uraniireducens rf4 *
51	B		*Brachyspira pilosicoli 95/1000 *
52	B		*Pseudogulbenkiania sp. nh8b *
53	B		*Dechloromonas aromatica rcb *
54	B		*Maribacter sp. htcc2170 *
55	B		*Zobellia galactanivorans *
56	B		*Escherichia coli bw2952 *
57	B		*Erwinia amylovora atcc 49946 *
58	B		*Gramella forsetii kt0803 *
59	B		*Klebsiella variicola at-22 *
60	B		*Salmonella enterica subsp. arizonae serovar *
61	B		*Yersinia enterocolitica subsp. enterocolitica 8081 *
62	B		*Methylomonas methanica mc09 *
63	B		*Borrelia turicatae 91e135 *
64	B		*Cronobacter turicensis z3032 *
65	B		*Yersinia pseudotuberculosis pb1/+ *
66	B		*Xanthomonas oryzae pv. oryzae maff 311018 *
67	B		*Tropheryma whipplei tw08/27 *
68	B		*Spirochaeta smaragdinae dsm 11293 *
69	B		*Sphingobacterium sp. 21 *
70	B		*Dyadobacter fermentans dsm 18053 *
71	B		*Eubacterium eligens atcc 27750 *
72	B		*Chlamydophila pneumoniae ar39 *
73	B		*Pelodictyon phaeoclathratiforme bu-1 *
74	B		*Desulfovibrio vulgaris str. hildenborough *
75	B		*Prosthecochloris aestuarii dsm 271 *
76	B		*Dinoroseobacter shibae dfl 12 *
77	B		*Acidiphilium cryptum jf-5 *
78	B		*Anaerolinea thermophila uni-1 *
79	B		*Thauera sp. mz1t *
80	B		*Magnetococcus sp. mc-1 *
81	B		*Sinorhizobium meliloti 1021 *
82	B		*Bordetella petrii dsm 12804 *
83	B		*Chloroflexus aggregans dsm 9485 *
84	B		*Corynebacterium glutamicum r *
85	B		*Cyanothece sp. pcc 7822 *
86	B		*Starkeya novella dsm 506 *
87	B		*Arcanobacterium haemolyticum dsm 20595 *
88	B		*Rhodopseudomonas palustris dx-1 *
89	B		*Rhodospirillum centenum sw *
90	B		*Xanthobacter autotrophicus py2 *
91	B		*Mycobacterium leprae br4923 *
92	B		*Gluconacetobacter diazotrophicus pal 5 *
93	B		*Streptomyces griseus subsp. griseus nbrc 13350 *
94	B		*Streptomyces scabiei 87.22 *
95	B		*Intrasporangium calvum dsm 43043 *
96	B		*Burkholderia rhizoxinica hki 454 *
97	B		*Haliangium ochraceum dsm 14365 *
98	B		*Salinibacter ruber m8 *
99	B		*Rothia dentocariosa atcc 17931 *
100	B		*Bifidobacterium animalis subsp. lactis ad011 *
